# Case study of an autoantibody panel for early detection of lung cancer and ground-glass nodules

**DOI:** 10.1007/s00432-020-03309-4

**Published:** 2020-07-09

**Authors:** Yunli Huo, Zijian Guo, Xuehui Gao, Zhongjuan Liu, Ruili Zhang, Xuzhen Qin

**Affiliations:** Peking Union Medical College Hospital, Peking Union Medical College and Chinese Academy of Medical Sciences, Shuaifuyuan 1#, Wangfujing, Dong Cheng, Beijing, 100730 People’s Republic of China

**Keywords:** Autoantibodies, Biomarkers, Lung cancer, Ground-glass nodules

## Abstract

**Purpose:**

Increasing lung cancer incidence in China with a high death rate due to late diagnosis highlights the need for biomarkers, such as panels of autoantibodies (AAbs), for prediction and early lung cancer diagnosis. We conducted a study to further evaluate the clinical performance of an AAb diagnostic kit.

**Methods:**

Using enzyme-linked immunosorbent assay, levels of seven AAbs in serum samples from 121 patients with newly diagnosed lung cancer, 84 controls (34 healthy individuals and 50 patients with benign lung disease), and 100 indeterminate solid nodules, were measured. Participants were followed up until 6 months after a positive test result to confirm lung cancer diagnosis.

**Results:**

The seven AAb concentration was significantly higher in lung cancer patients than in controls (*P* < 0.05). The seven AAb sensitivity and specificity for newly diagnosed lung cancer were 45.5% and 85.3%, respectively, while the seven AAb combined area under the curve (in lung cancer patients versus controls) was 0.660. Of the 28 patients with solid nodules with positive test results, 8 and 3 were diagnosed with lung cancer and benign lung disease, respectively, during follow-up. The positive predictive value of the experiment was 72.7%.

**Conclusion:**

Positive AAb test results were associated with a high risk of lung cancer. The seven-AAb panel also had a high predictive value for detecting lung cancer in patients with solid nodules. Our seven lung cancer autoantibody types can provide an important early warning sign in the clinical setting.

## Background

Lung cancer has the highest mortality rate among all common cancer types, and it has shown a trend of increasing incidence worldwide (She et al. 2013). According to statistics from the American Cancer Association, there were 226,160 patients with newly diagnosed lung cancer in 2012, while 160,340 people died from lung cancer in 2012 (Siegel et al. 2012). In China, 4.292 million people were diagnosed with lung cancer, and there were 2.814 million deaths due to lung cancer in 2015 (Chen et al. 2016). Early detection is the key to long-term survival, as the 5-year survival rate drops significantly with each cancer stage. For example, the survival rate for stage 0 lung cancer is more than 90% and that for stage I lung cancer is more than 60%, whereas it drops from 40% to 5% as stage II disease progresses to stage IV disease (Wei 2010). Despite the advances in treatment over the years, there has been little change in the 5-year survival rate in recent years. At present, most lung cancers are still diagnosed at a late stage, which leads to the high mortality rate.

The current approach to detect lung cancer relies mainly on imaging techniques such as X-ray, computed tomography (CT), and magnetic resonance imaging (MRI) (Baykul et al. 2010; Kaur et al. 2012). The low sensitivity or specificity of these techniques has contributed to their ineffectiveness in detecting lung cancer at an early stage (Jemal et al. 2004). Autoantibodies (AAbs) have been explored as promising biomarkers to detect lung cancer at an early stage (Ren et al. 2017). In this study, we aimed to use the seven AAbs’ (GAGE7, CAGE, MAGEA1, SOX2, GBU4-5, PGP9.5, and p53) combination detection methods, especially for patients with suspected lung cancer, focusing on lung nodules in the patients. This can be combined with computed tomography (CT) for an early prediction of lung cancer and reduction in lung cancer mortality among patients.

## Materials and methods

### Study participants

We enrolled 121 patients who were newly diagnosed with lung cancer at Beijing Union Medical College Hospital from September 2017 to March 2018. There were 61 men and 60 women (median age, 60 years). In addition, we enrolled 34 healthy individuals, 50 patients with benign lung disease, and 100 patients with suspected lung cancer (Table 1). We collected patient information such as sex, age, smoking history, clinical stage, histological location, pathology, and presence of lung nodules.

**Table 1:** General characteristics of the study participants

Characteristic	All participants	Patients with lung cancer	Healthy participants	Patients with benign lung disease	Patients with suspected lung cancer	*P *value
	*N* = 305	*N* = 121	*N* = 34	*N* = 50	*N* = 100	
Average age (years)	59.5	60.6	59.5	63 .1	58.7	> 0.05
Sex, *n* (%)						> 0.05
Male	145 (47.54%)	61 (50.4%)	15 (44.1%)	26 (52.0%)	43 (43.0%)	
Female	160 (52.46%)	60 (49.6%)	19 (55.9%)	24 (48.0%)	57 (57.0%)	
Smoking history, *n* (%)						> 0.05
Ever/current		42 (34.7%)	9 (26.5%)	19 (38.0%)	37 (37.0%)	
Never		79 (54.3%)	25 (73.5%)	31 (62.0%)	63 (63.0%)	
Histological type, *n* (%)						
Small cell lung cancer		2 (1.7%)				
Squamous cell carcinoma		14 (11.6%)				
Squamous adenocarcinoma		1 (0.8%)				
Adenocarcinoma		104 (85.9%)				
Large cell carcinoma		0 (0%)				
T stage, *n* (%)						
0		3 (2.5%)				
I		69 (57.0%)				
II		12 (9.9%)				
III		14 (11.6%)				
IV		23 (19.0%)				
Location of the tumor, *n* (%)						
Left lung		51 (42.1%)				
Right lung		63 (52.0%)				
Both lungs		2 (1.7%)				
Unknown		5 (5.9%)				

*SD* standard deviation

### Participant selection

#### Inclusion criteria for patients with lung cancer

(1) Patients who did not undergo chemotherapy, radiotherapy, surgery, and other interventions prior to the study; (2) patients with a confirmed diagnosis of lung cancer (based on clinical symptoms and X-ray, lung CT, MRI, and other radiological examination results; fiberoptic bronchoscopy biopsy; brush biopsy; and pathological analysis of exfoliated sputum and pleural fluid cells), according to the 2017 guidelines on the diagnosis and treatment of primary lung cancer; (3) patients without other tumors or secondary lung metastasis; (4) patients with complete medical history; and (5) patients without autoimmune disease.

#### Inclusion criteria for healthy individuals

(1) Individuals with apparently normal imaging findings (CT, B-mode ultrasonography) and (2) individuals with normal blood routine and biochemical test results.

#### Inclusion criteria for patients with benign lung disease

(1) Patients with a confirmed diagnosis of benign lung disease at our hospital or other hospitals. In the clinical department of Peking Union Medical College Hospital, benign lung diseases are defined as non-lung cancer diseases, including infectious lung disease, emphysema, chronic obstructive emphysema, occupation-related lung disease, immune-related lung disease, genetic lung disease, alveolar proteinosis, and alveolar microlithiasis.

#### Inclusion criteria for patients with suspected lung cancer (pulmonary nodules)

(1) Patients with clinically undiagnosed lung nodules according to the National Comprehensive Cancer Network Lung Cancer Screening Guidelines.

#### Exclusion criteria

Individuals who did not meet the above-listed inclusion criteria.

### Specimen collection and treatment

All participants (including patients and healthy individuals) were requested to fast in the morning, and a 3–5 mL sample of venous blood was collected using a vacuum blood collection tube containing a separation gel. The sample was centrifuged at 3000 rpm for 10 min, and the serum was separated. Samples that could not be processed immediately were stored at − 80 °C for future testing.

### Main reagents and instruments

We measured the levels of the seven AAbs using enzyme-linked immunosorbent assay (ELISA)-based test kit purchased from Hangzhou Cancer Probe Biological Technology Co., Ltd., Room 501–505, 5th Floor, Building D, Building 688, Bin Anlu, Changhe Street, Binjiang District, Hangzhou, China (registration certificate number: National Machinery Note Standard 20153402087; kit for qualitative detection of seven AAbs [p53, GAGE7, PGP9.5, CAGE, MAGEA1, SOX2, and GBU4-5]). According to the manufacturer’s instructions, we diluted the serum 100 times and applied 100 μL of sample per well. The instrument used for detection was the Addcare ELISA 600 automatic enzyme-free analyzer (Addcare, Produced by Yantai Abcon Company China). Absorbance was measured at 450 nm. The reference ranges (U/mL) for the AAbs are as follows: p53 < 7.0, PGP9.5 < 20.0, SOX2 < 7.0, GAGE7 < 5.4, GBU4-5 < 7.0, MAGEA1 < 10.0, and CAGE < 6.0.

### Statistical analysis

Sensitivity and specificity were calculated using the equations provided below:

$$ {\text{Sensitivity}}\left( \% \right) = a/\left( {a \, + \, c} \right) \, \times \, 100. $$

$$ {\text{Specificity}}\left( \% \right) = d/\left( {b + d} \right) \times 100. $$

$$ {\text{Positive predictive value }}\left( {{\text{PPV}}} \right)\left( \% \right) = a/\left( {a + b} \right) \times 100. $$

$$ {\text{Negative predictive value }}\left( {{\text{NPV}}} \right)\left( \% \right) = d/\left( {c + d} \right) \times 100. $$

$$ {\text{Precision }}\left( \% \right) = \left( {a + d} \right)/\left( {a + b + c + d} \right) \times 100. $$

‘*a*’ is the number of samples that tested positive for the seven AAbs by the test kit and the reference method (true positives), ‘*b*’ is the number of samples that tested positive by the test kit but negative by the reference method (false positives), ‘*c*’ is the number of samples that tested negative by the test kit but positive by the reference method (false negatives), and ‘*d*’ is the number of samples that tested negative by both methods (true negatives).

Statistical analyses and graphing were performed using IBM SPSS Statistics for Windows, version 22.0 statistical software (IBM Corp., Armonk, NY, USA). *P* values < 0.05 were considered statistically significant.

## Results

We recruited a total of 305 participants. Their characteristics are displayed in Table 1. In the lung cancer group, there were 61 men (50.4%) and 60 women (49.6%); among them, 42 (34.7%) had a history of smoking. In the suspected lung cancer group, there were 43 men (43.0%) and 57 women (57.0%); among them, 37 (37.0%) had a history of smoking.

Using the collected data, we conducted a detailed analysis of lung cancer patients (Table 2) and suspected lung cancer patients (Table 3). Combined with seven autoantibody targets, the positive rate of detection was not affected by the lung cancer subtype and TNM stage (*P* > 0.05) (Tables 2, 3). In the suspected lung cancer group, the positive rate of detection of the seven AAbs was not affected by tumor subtype, TNM stage, or smoking history (*P* > 0.05) (Table 3).

**Table 2:** Comparison of the positive rate of the combined detection of the seven autoantibodies in patients with lung cancer with different clinicopathological characteristics

Characteristic	Total number of cases	Positive cases,* n*	Positive cases, %	p53	PGP9.5	SOX2	GAGE7	GBU4-5	MAGEA1	CAGE
T stage, *n* (%)										
0	3	2	66.67%	4.93	2.37	4.31	3.91	4.74	4.17	2.52
I	69	29	42.03%	3.76	2.01	2.59	4.87	2.13	2.34	3.03
II	12	5	41.67%	6.45	2.38	1.94	7.74	1.98	2.23	6.80
III	14	5	35.71%	2.38	1.93	2.95	3.90	1.22	2.20	2.79
IV	23	10	43.48%	3.49	1.48	3.80	2.93	1.44	2.39	4.66
*P* value			0.915	0.289	0.975	0.635	0.728	0.527	0.95	0.254
Smoking history, *n* (%)										
Ever/current	42	17	40.48%	2.93	1.79	2.65	6.10	1.73	2.13	3.28
Never	79	34	43.04%	4.33	2.03	2.94	3.87	2.06	2.50	3.90
*P* value			0.58	0.019	0.183	0.323	0.084	0.095	0.266	0.308
Histological type, *n* (%)										
Adenocarcinoma	104	44	42.31%	3.91	1.85	2.75	3.94	2.07	2.46	3.69
Squamous cell carcinoma	14	5	35.71%	3.54	3.02	3.10	7.69	1.06	2.13	3.74
Small cell lung cancer	2	2	100.00%	3.93	0.22	7.05	21.68	2.12	0.35	4.16
Squamous adenocarcinoma	1	0	0.00%	3.84	1.94	2.84	4.65	1.94	2.37	3.68
*P* value			0.3	0.903	0.667	0.471	0.04	0.762	0.814	0.955

*T* tumor

**Table 3:** Comparison of the positive rate of the combined detection of the seven autoantibodies in patients with suspected lung cancer with different clinicopathological characteristics

Characteristic	Total number of cases	Positive cases, n	Positive cases, %	p53	PGP9.5	SOX2	GAGE7	GBU4-5	MAGEA1	CAGE
Smoking history, *n*										
Ever/current	37	10	27.03%	1.43	0.94	1.77	1.66	1.9	1.61	1.13
Never	63	18	28.57%	3.73	0.69	1.47	1.44	3.16	1.68	1.33
*P* value			0.741	0.017	0.404	0.451	0.476	0.171	0.957	0.674
Histological type, *n* (%)										
Benign disease	6	3	50%	0.7	2.05	0.8	2.08	6.21	0.71	0.44
Adenocarcinoma	16	6	37.5%	2.97	0.53	3.45	5.3	0.77	4.23	3.39
Squamous cell carcinoma	3	0	0%	1.95	0.74	1.85	0.38	1.95	0.92	1.21
Small cell lung cancer	2	1	50%	2.06	0.27	6.8	0.26	0.27	0.23	0.45
Other subtypes of lung cancer	2	1	50%	0.49	0.17	0.4	0.52	4.67	0.18	0.66
*P* value			0.698	0.951	0.765	0.625	0.715	0.146	0.802	0.817
T stage, *n*										
I	15	3	20%	0.96	0.56	1.62	2.83	0.31	0.35	0.44
II	2	1	50%	2.51	1.02	7.01	0.48	1.65	0.30	0.19
III	2	1	50%	1.20	0.08	5.49	7.32	1.36	5.44	12.56
IV	2	1	50%	16.92	0.18	2.78	2.22	2.93	17.35	2.65
*P* value			0.648	0.016	0.926	0.421	0.84	0.015	0.014	0.008

*T* tumor

**Fig. 1:**
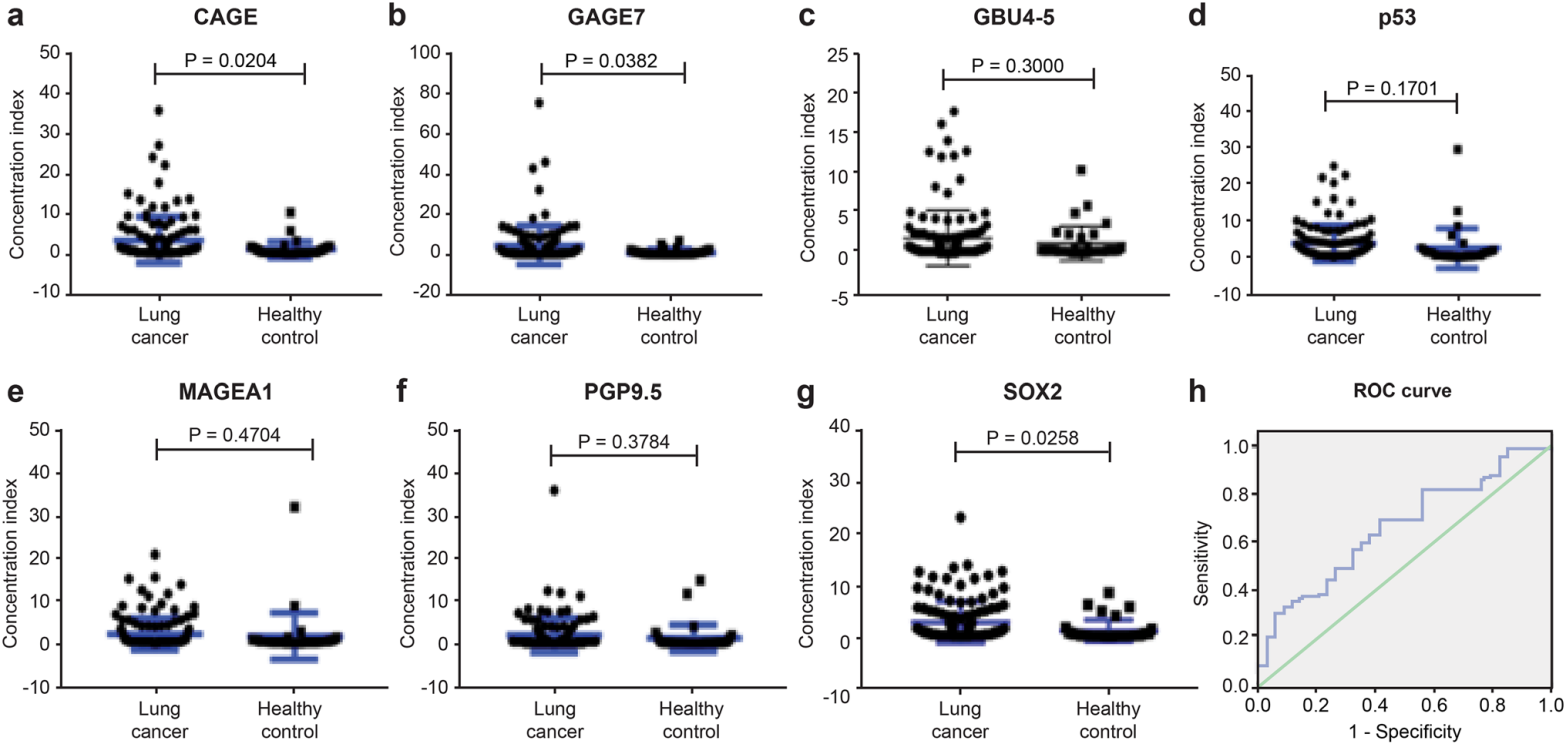
**a**–**g** Quantification of serum levels of each of the seven autoantibodies in patients with lung cancer (*n* = 121) and healthy controls (*n* = 34) (mean ± s.e.m., unpaired student’s *t *test). **h** Performance of the seven-autoantibody panel by the receiver operating characteristic (ROC) curve analysis in the lung cancer cohort (*n* = 121) and controls (*n* = 84); the green line indicated is the diagonal line without discrimination while the blue line is the ROC line. The ROC curves were constructed by calculating the sensitivity/specificity of the test for a succession of deviations from the original cut-offs, with the same deviation for each antigen in the panel

**Fig. 2:**
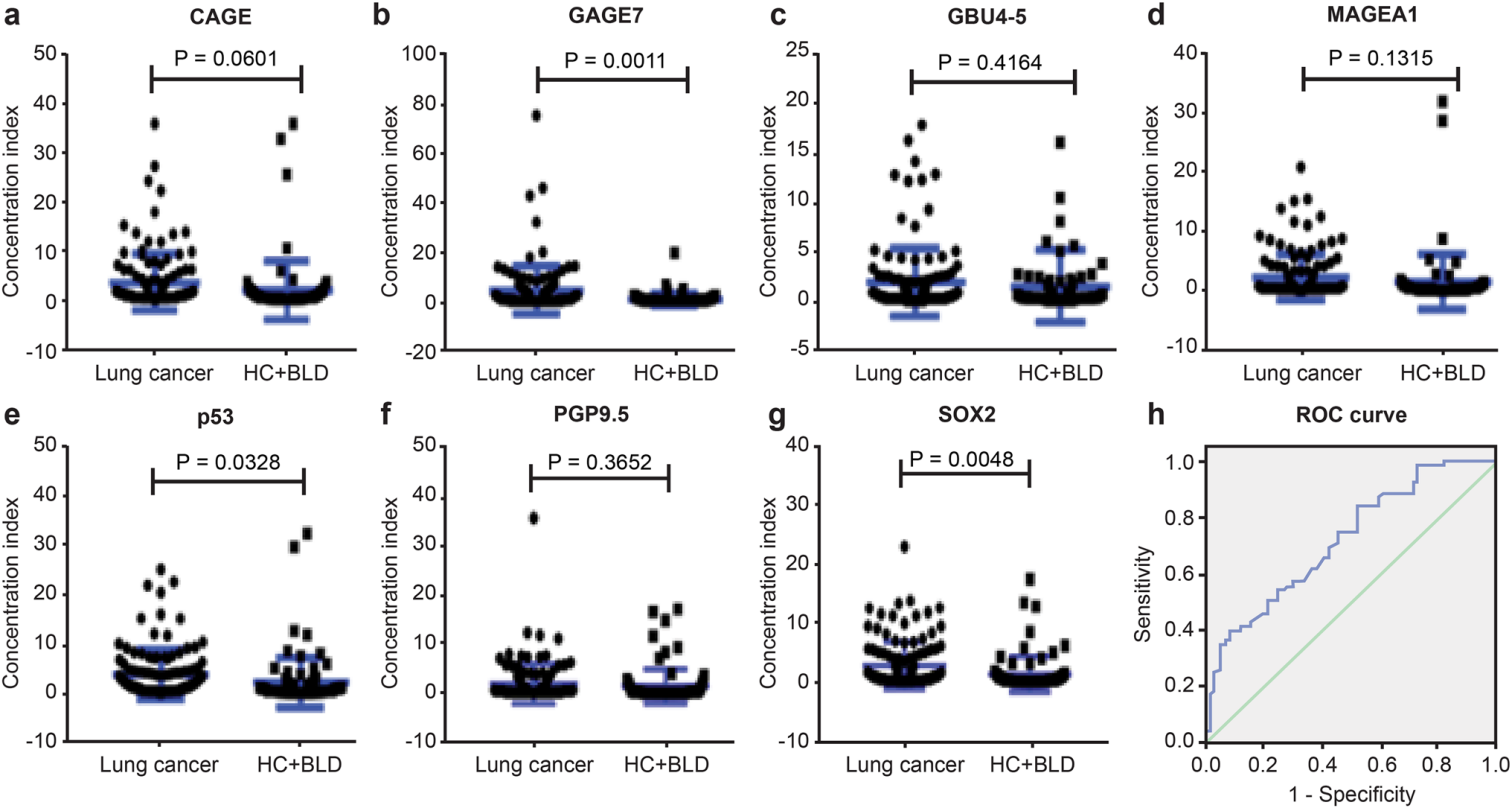
**a**–**g** Quantification of serum levels of each of the seven autoantibodies in patients with lung cancer (*n* = 121) and the non-malignant control group (*n* = 84). These include healthy controls (HC) and patients with benign lung disease (BLD) (mean ± s.e.m., unpaired student’s *t *test). **h** Performance of the seven-autoantibody panel by the receiver operating characteristic (ROC) curve analysis in the lung cancer cohort (*n* = 121) and controls (*n* = 84); the green line indicated is the diagonal line without discrimination while the blue line is the ROC line. The ROC curves were constructed by calculating the sensitivity/specificity of the test for a succession of deviations from the original cut-offs, with the same deviation for each antigen in the panel

The concentrations of the seven AAbs (p53, GAGE7, PGP9.5, CAGE, MAGEA1, SOX2, and GBU4-5) were measured by ELISA in 205 serum samples (lung cancer, *n* = 121; benign lung disease, *n* = 50; healthy controls, *n* = 34). As shown in Fig. 1, the individual values for CAGE, GAGE7, and SOX2 differed significantly between the lung cancer and healthy control groups (*P* = 0.02, 0.04, and 0.03, respectively). However, the values for GBU4-5, p53, MAGEA1, and PGP9.5 did not differ significantly between the two groups. The test result was determined as positive when at least one AAb had a score deemed as positive. The sensitivity of the assay was 45.5% when the control group only included healthy participants [95% confidence interval (CI) 38–56%], and the specificity was 85.3% (95% CI 73–95%). The assay performed similarly when the control group included both healthy participants and participants with benign lung disease. As shown in Fig. 2, the individual values for p53, GAGE7, and SOX2 differed significantly between the lung cancer and control groups consisting of healthy participants and those with benign lung disease (*P* = 0.03, 0.001, and 0.005, respectively). However, the values for GBU4-5, CAGE, MAGEA1, and PGP9.5 did not show significant differences. The sensitivity was 45.5% (95% CI 38–56%) and the specificity was 83.3% (95% CI 75–94%). Based on receiver operating characteristic (ROC) curve analysis, the combined area under the curve (AUC) of all seven AAbs in the lung cancer versus control groups (healthy participants plus patients with benign lung disease) was 0.660 (Table 4).

**Table 4:** Diagnostic accuracy of the panel consisting of seven autoantibodies used to screen for lung cancer

Criterion for positive screening for lung cancer	Sensitivity (patients with lung cancer)	Specificity (healthy individuals)	Specificity (patients with benign lung disease)	Specificity (healthy individuals and patients with benign lung disease)
	*N* = 121	*N* = 34	*N* = 50	*N* = 84
1 strongly positive AAb test result	55/121 (45.5%, 95% CI 38–56%)	29/34 (85.3%, 95% CI 73–95%)	–	70/84 (83.3%, 95% CI 75–94%)
≥ 1 strongly positive and ≥ 2 moderately positive AAb test results	67/121 (55.4%)	29/34 (85.3%)	–	70/84 (83.3%)
≥ 2 moderately positive AAb test results	12/121 (9.9%)	34/34 (100%)	50/50 (100%)	–

*AAb* autoantibody, *CI* confidence interval

**Fig. 3:**
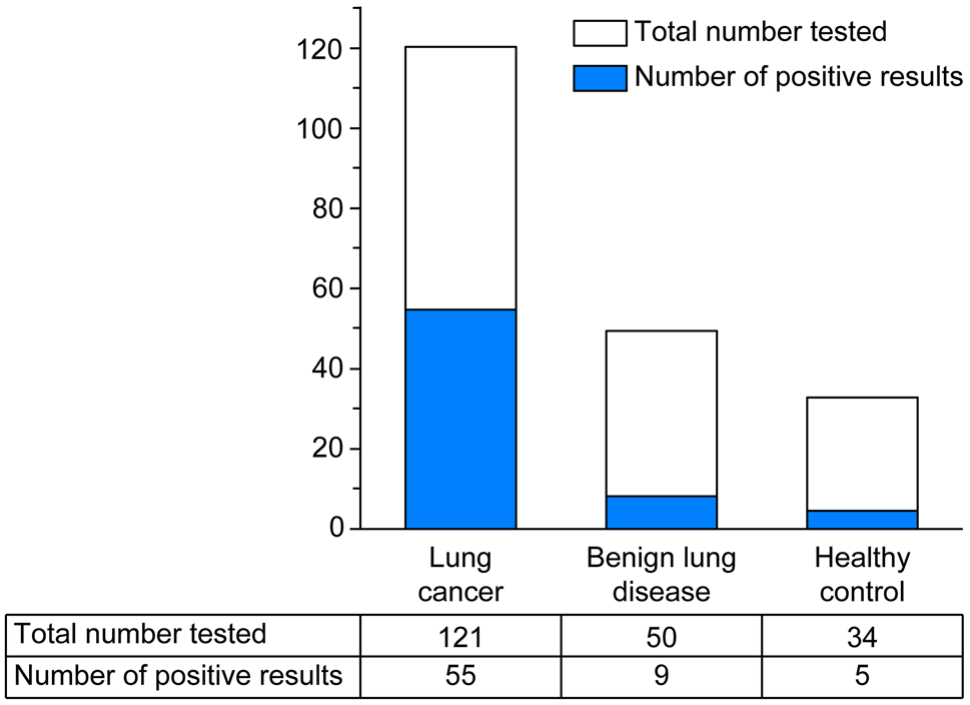
The positive rates of detection of the seven autoantibodies in patients with lung cancer, patients with benign lung disease, and healthy controls

Figure 3 shows the results of the seven AAb tests in the lung cancer and non-lung cancer groups. Of the 121 participants in the lung cancer group, 55 (45.5%) tested positive; of the 50 patients with benign lung disease, 9 (18%) tested positive; and of the 34 healthy participants, 5 (14.7%) tested positive.

To achieve high specificity based on a panel of seven AAbs, high cutoff values were assigned for each AAb, leading to lower sensitivity of detection. A second set of lower cutoff values was introduced such that medium levels of AAbs could be considered as “medium positive”. These concentrations fell between the high and medium cutoff values. The presence of “medium positive” results for at least two AAbs was considered a positive test result for the seven-AAb panel. If there was at least one “strongly positive” result and at least two “medium positive” results, the test result was considered positive. The assay sensitivity increased to 55.4% at a specificity of 85.3%, without sacrificing the specificity. Based on the ROC curve analysis, the combined AUC of all seven AAbs in the lung cancer group versus the non-lung cancer control group (healthy participants plus patients with benign lung disease) was 0.719 (Table 4).

**Fig. 4:**
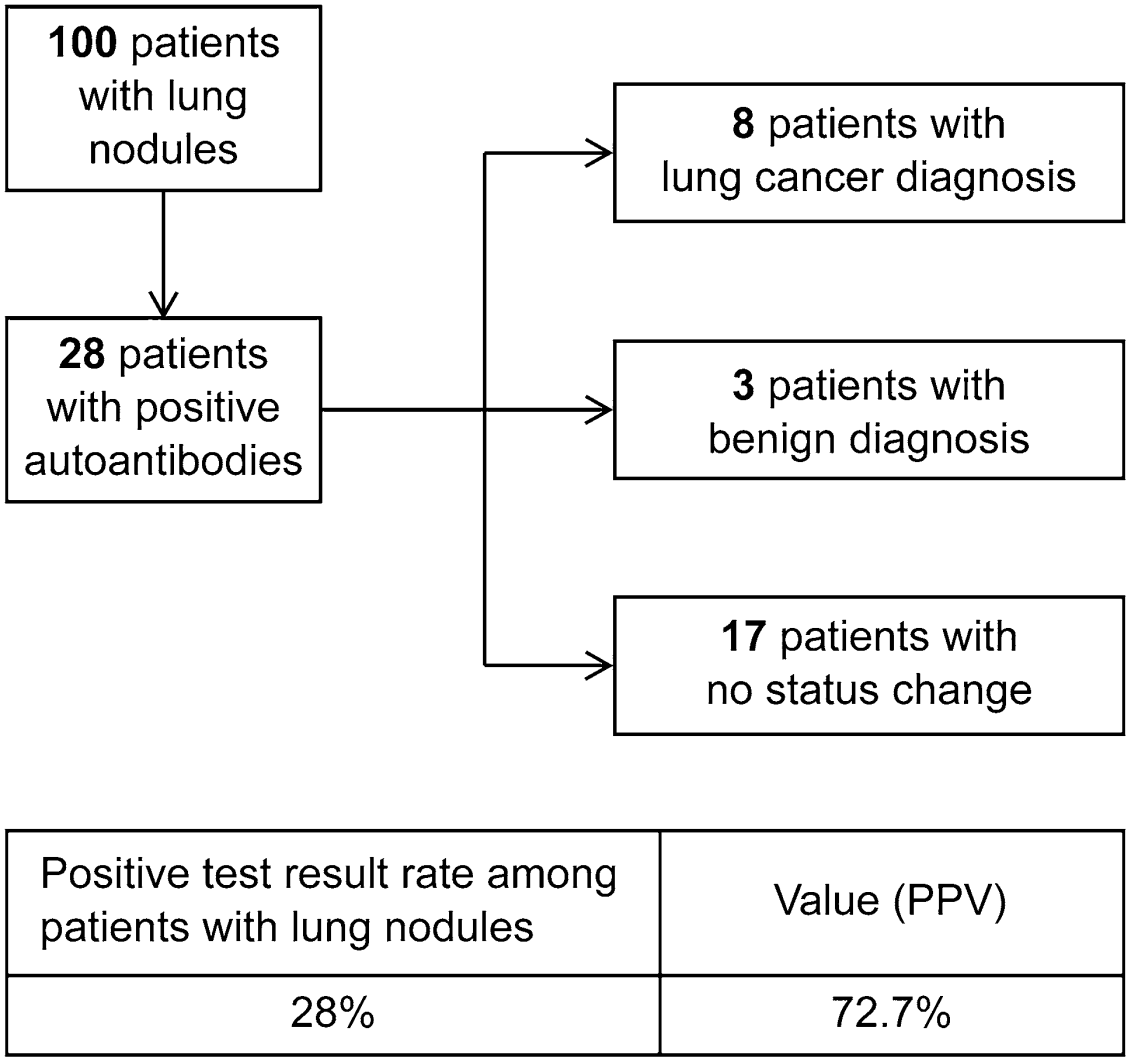
Follow-up data of patients with pulmonary nodules

This study also included 100 patients who presented with GGNs and/or nodules. A flowchart of these patients is shown in Fig. 4. The positive rate of AAbs among patients with lung nodules was 28% (28/100). To evaluate the clinical value of the seven-AAb panel in the early detection of lung cancer, we evaluated the updated patient medical records of these 28 subjects. After approximately 6 months, 17 of the 28 patients did not show any changes in the medical diagnosis pertaining to the lung. Of the remaining 11 patients, 8 were subsequently diagnosed with lung cancer and three were diagnosed with benign lung disease. Although these results are preliminary, the PPV of the seven-AAb panel was 72.7%.

## Discussion

Our study showed that the combined AAb panel (p53, GAGE7, PGP9.5, CAGE, MAGEA1, SOX2, and GBU4-5) could differentiate patients with lung cancer from healthy controls and patients with benign lung disease. This is consistent with the results of a previous study (Chapman et al. 2012). The level of each AAb was not significantly correlated with TNM tumor stage in the lung cancer and suspected lung cancer groups. This is consistent with the results obtained by Healey et al. (2013), who reported that AAb levels are not associated with lung cancer stage.

One important observation from this study is that the interpretation of results is the key to understanding the performance of the combined AAb panel. To achieve high specificity of the seven-AAb panel, the cutoff values were set such that the specificity of each AAb ranged from 95 to 99%. As immune responses toward the same antigen could vary greatly among the population due to the heterogeneity in genetics and environmental factors, a weaker immune response toward the same level of tumor antigen is not correlated with a lower probability of tumor formation. We also reason that the presence of more than one immunoreactive AAb response leads to an increase in the probable risk of cancer. Accordingly, we found that if two or more “medium positive” AAb responses were considered positive, the sensitivity of lung cancer detection increased, without sacrificing the specificity. This observation might help clinicians interpret AAb panel results in a hospital setting.

To further evaluate the clinical value of the seven-AAb panel in the early detection of lung cancer, we collected specimens from 100 patients who presented with GGNs or lung nodules without any further diagnosis of lung cancer. For an interim analysis, we collected the clinical information of 28 subjects with positive AAb results. Even though only 6 months had elapsed following the initial tests, eight patients were diagnosed with lung cancer while three were diagnosed with benign lung disease. Based on these data, the PPV of the seven-AAb panel was 72.7%. We expect a better estimate of the PPV from ongoing studies with a longer follow-up period. The seven-AAb panel showed positive results in patients with lung nodules who were not diagnosed with lung cancer by other clinical means, which provides a certain early warning sign. Combined with low-dose CT (LDCT) findings, this information could add clinical value for the early discrimination of benign and malignant lung nodules.

Lung cancer is one of the most common cancers worldwide and the main cause of cancer-related deaths (He et al. 2018; Chen et al. 2016; Miller et al. 2016). The mean 5-year survival rate for lung cancer is 17.4%, but the survival rate declines as the cancer becomes more advanced; for example, the 5-year survival rate in patients with metastatic lung cancer is only 4.2% (Cronin et al. 2018), while the 10-year survival rate in patients with stage Ia lung cancer may be as high as 92% (International Early Lung Cancer Action Program Investigators et al. 2006). As 85% of patients with lung cancer are not diagnosed until the cancer is clinically advanced, these patients have a poor quality of life. Currently, the medical community is focusing on the early diagnosis of lung cancer to improve overall survival (Palma et al. 2016). LDCT is currently the method of choice to screen for lung cancer in high-risk patients and is recommended by the American medical community (Ruchalski et al. 2016; Humphrey et al. 2013). LDCT has high sensitivity; however, it has a false-positive rate of up to 96.4% when used for screening (National Lung Screening Trial Research Team et al. 2011; Boiselle 2013) and is associated with inevitable radiation risks. Therefore, we used serum samples to screen for seven types of AAbs associated with lung cancer. Although the AAb tests were not as sensitive as LDCT, they had relatively high specificity. Additionally, they had a high PPV in our study participants. We hope to use these seven AAbs to screen for early lung cancer. We can screen patients with newly diagnosed multiple pulmonary nodules with LDCT in combination with serum AAb testing to provide reliable clinical monitoring data.

This study had some limitations. One drawback was the small number of samples; second, this study was conducted in only one center. Further research is warranted. We intend to increase the number of patients with pulmonary nodules in follow-up multicenter and regional studies, to reduce the effect of small sample sizes on the results.

In conclusion, using AAb testing in combination with LDCT could improve its PPV and lower the cumulative radiation risk posed by repeated LDCT scans. The presence of seven different AAbs related to lung cancer (GAGE7, CAGE, MAGEA1, SOX2, GBU4-5, PGP9.5, and p53) was analyzed in different populations, and the study demonstrated the clinical value of providing a reliable basis for early laboratory diagnosis in patients with early lung cancer. In future, we plan to continue with our study on the autoantibodies of lung cancer patients with increased number of patients with lung nodules, to reduce the difficulty in detecting lung cancer or in identifying lung autosarcoma in patients, using the combination of autoantibodies in the clinical setting. We also hope to provide laboratory aids for early diagnosis of lung cancer to reduce the mortality of lung cancer in patients, thus, increasing the patients’ quality of life.
